# Familial Partial Lipodystrophy: Clinical Features, Genetics and Treatment in a Greek Referral Center

**DOI:** 10.3390/ijms241512045

**Published:** 2023-07-27

**Authors:** Aikaterini Kountouri, Emmanouil Korakas, Eirini Maratou, Ignatios Ikonomidis, Konstantinos Balampanis, Stavros Liatis, Nikolaos Tentolouris, Panagiotis Toulas, Foteini Kousathana, Christophoros Giatzakis, George D. Dimitriadis, Vaia Lambadiari

**Affiliations:** 1 Second Department of Internal Medicine, Medical School, National and Kapodistrian University of Athens, Attikon University Hospital, 12462 Athens, Greece; katerinak90@hotmail.com (A.K.); f.kousathana@hotmail.com (F.K.); 2 Laboratory of Clinical Biochemistry, Medical School, National and Kapodistrian University of Athens, Attikon University Hospital, 12462 Athens, Greece; maratou@hotmail.com; 3 Laboratory of Preventive Cardiology, Second Cardiology Department, Medical School, National and Kapodistrian University of Athens, Attikon University Hospital, 12462 Athens, Greece; 4 First Department of Propaedeutic and Internal Medicine, Medical School, National and Kapodistrian University of Athens, Laiko General Hospital, 11527 Athens, Greecententolouris@yahoo.gr (N.T.); 5 Bioiatriki Healthcare Group, 11526 Athens, Greece; 6 DNAbiolab, Cretan Center for Research and Development of Applications on Genetics and Molecular Biology, 71306 Heraklion, Greece; 7 Sector of Medicine, Medical School, National and Kapodistrian University of Athens, 15772 Athens, Greece; gdimitr@med.uoa.gr

**Keywords:** lipodystrophy, diabetes mellitus, FPLD, metreleptin, exons, introns, hypertriglyceridemia, insulin resistance

## Abstract

Familial partial lipodystrophy (FPLD) is a rare syndrome in which a patient’s phenotype is not merely dependent on the specific genetic mutation, but it is also defined by a combination of other demographic, environmental and genetic factors. In this prospective observational study in a Greek referral center, we enrolled 39 patients who fulfilled the clinical criteria of FPLD. A genetic analysis was conducted, which included sequence and deletion/duplication analyses of the LMNA and PPRARG genes, along with anthropometric and metabolic parameters. The treatment responses of patients who were eligible for treatment with metreleptin were evaluated at 3 and 12 months. In most of the patients, no significant changes were detected at the exon level, and any mutations that led to changes at the protein level were not associated with the lipodystrophic phenotype. On the contrary, various changes were detected at the intron level, especially in introns 7 and 10, whose clinical significance is considered unknown. In addition, treatment with metreleptin in specific FPLD patients significantly improved glycemic and lipidemic control, an effect which was sustained at the 12-month follow-up. More large-scale studies are necessary to clarify the genetic and allelic heterogeneity of the disease, along with other parameters which could predict treatment response.

## 1. Introduction

Lipodystrophies constitute a group of rare, heterogeneous disorders, which are characterized by a partial or generalized deficiency of adipose tissue [1]. Depending on the underlying mechanism, they are classified as congenital or acquired, while depending on the distribution of the fat loss, they are further divided into generalized or partial. The estimated prevalence of the disease is 1.3–4.7 cases per million people, although it is evident that these numbers do not reflect the real picture, as these syndromes are often underdiagnosed. Apart from anatomical abnormalities, lipodystrophies are often accompanied by severe metabolic derangement, which includes insulin resistance, diabetes mellitus, hypertriglyceridemia, non-alcoholic fatty liver disease and premature cardiovascular disease [2,3].

Familial partial lipodystrophy (FPLD) is a form of congenital lipodystrophy. The loss of adipose tissue primarily affects the limbs and hips, while fat in the areas of the head, neck and trunk is preserved or even excessive, although the disease is characterized by significant phenotypic variability [4]. Its prevalence has been estimated to be around one in one hundred thousand. There are six main types of the disease (FPLD1 to FPLD6), of which FPLD2 (Dunnigan syndrome) and FPLD3 are the most common, affecting more than 500 patients and 20 families, respectively. Both types follow an autosomal dominant inheritance pattern, with the genes responsible being the LMNA gene (lamin A/C) on chromosome 1q22 for FPLD2 (OMIM#: 151,660) and the peroxisome proliferator-activated receptor gamma (PPARG) gene on chromosome 3p25.2 for FPLD3 (OMIM#: 604,367) [5]. Together, the LMNA and PPARG variants are responsible for about half of all recognized cases of FPLD. Other genetic abnormalities include mutations in genes such as PLIN1, CIDEC and AKT2, among others, which account for several extremely rare types of the disease with scarce case reports in the literature. LMNA pathogenic variants are generally considered to cause more significant adipose tissue loss compared to PPARG variants; however, accumulating evidence in recent years has shown that a patient’s phenotype is not merely dependent on the specific genetic mutation, but it is also defined by a combination of other demographic (age, gender), environmental and genetic factors, such as single-nucleotide variants (SNVs) and DNA methylation, which can alter the eventual phenotypic expression [6].

Until a decade ago, FPLD treatment was based only on lifestyle modifications, including strict dietary regimens and exercise, along with the commonly prescribed medications for the cardiometabolic comorbidities of the disease, with poor results. Metreleptin, a recombinant analogue of human leptin, was the first disease-specific medication which was approved by U.S. Food and Drug Administration (FDA) in 2014 for adults and children aged ≥12 years with FPLD for whom standard-of-care treatment had failed to achieve adequate control of the disease [7,8]. Although, due to the rarity of the disease, data from large-scale randomized clinical trials (RCTs) cannot be obtained, many studies and clinical cases have shown the beneficial effects of metreleptin on aspects such as glycemic control, insulin requirements, triglyceride levels and albuminuria [9,10].

In this report, we present a large group of novel mutations in FPLD patients from a large referral center in Greece for whom genetic testing was conducted, and we discuss the possible relationship between the genetic variations detected and their phenotype. As lipodystrophy is a rare disease and data about the effect of specific genotypes on the clinical manifestations are scarce, patient registries could help to shed light on the pathophysiological, biochemical and molecular mechanisms of the disease. The clinical characteristics of the patients eligible for treatment with metreleptin are thoroughly described, along with the results of the metreleptin therapy during the subsequent follow-up.

## 2. Results

### 2.1. Demographic Characteristics 

Overall, 39 patients were identified to fulfill the criteria of having FPLD. The mean ± SD age was 47.59 ± 10.13 years, and 30 patients were female (76.9%).

### 2.2. Clinical Characteristics

The clinical characteristics of the study participants at the baseline visit are summarized in Table 1. Regarding the reason for their medical consultations, twenty (51.3%) patients were referred to our center due to uncontrolled T2DM, five (12.8%) due to uncontrolled T1DM, one (2.6%) due to gestational diabetes mellitus, nine (23.1%) due to NASH and five (12.8%) due to hypertriglyceridemia. All the patients presented lipoatrophy in their lower limbs, 23 patients (59%) in their upper limbs and 26 patients (66.7%) in their gluteal area. Thirty-eight patients (97.4%) displayed fat deposition in the abdomen, eighteen patients (46.2%) in the trunk, twenty-two patients in the face (56.4%) and fourteen patients (35.9%) in the neck. Thirty (76.9%) patients presented acanthosis nigricans while five (12.8%) patients displayed muscular hypertrophy. Three (10%) females presented hirsutism. Regarding the comorbidities related to the lipodystrophy phenotype, twenty-six patients (66.7%) suffered from diabetes mellitus, thirty patients (76.9%) presented dyslipidemia, twenty-four patients (61.5%) had hypertension, twenty-two patients (56.4%) had NASH or NAFLD and six patients (15.4%) suffered from heart diseases. Nine females (33.3%) met the criteria for PCOS.

### 2.3. Anthropometric and Biochemical Characteristics

The anthropometric and biochemical characteristics of the patients at the baseline visit are summarized in Table 2.

### 2.4. Genetic Analysis

The genetic analysis of the LMNA gene of the study participants is summarized in Table 3. The genetic analysis of the LMNA gene revealed that five (12.8%) patients present nucleotide changes in the introns of the LMNA gene, thirteen (33.3%) patients in the exons and five (12.8%) patients in both the introns and exons. Specifically, eight patients present nucleotide changes in intron 7. Fourteen patients carry synonymous nucleotide changes in exon 10 of the LMNA gene. Four patients present a nucleotide change in exon 5 and five in exon 7. In total, two patients likely have pathogenic mutations in the LMNA gene and one in the PPARG gene. Specifically, patient 24 presents a mutation (c.1969-1G > A) in intron 11, and patient 30 has a heterozygous mutation from G to T at nucleotide 786 in exon 4 of the LMNA gene which causes a substitution from glutamic acid to aspartic acid.

Patient 27 was found to carry out a novel heterozygous mutation in the PPARG gene (c470A>G, p. Glu157Gly, exon3) (The University of Chicago Genetic Services Laboratory). Τhe substitution of the negatively charged glutamic acid at position 157 by the hydrophobic glycine could possibly lead to the formation of a non-functional protein responsible for the lipodystrophy phenotype. According to the classification of the Missense Interpretation by Experimental Response (MITER) database, the described mutation is associated with a 78.6% probability of causing FPLD3 and a 6.5-fold increased risk for type 2 diabetes mellitus.

### 2.5. Alternative Splicing

In P1, we investigated the existence of alternative splicing. In all cases, a main amplimer was observed at the expected (normal) size both in the sample and control RNAs. The direct sequencing of this amplimer confirmed that it corresponds to the normal (non-alternatively spliced) product of the gene. In the cases in which the primer pairs 5F-10R and 5F-11R were used, several other amplimers were observed in very small quantities compared to the main amplimer. Attempts to directly sequence the amplimers from the primer pair 5F-10R failed due to very low quantities of the PCR products. Attempts to directly sequence the amplimers generated from the primer pair 5F-11R were partially successful. The amplimer of around 500 bp from random hexamer-generated cDNA consisted partly of exon 6 (up to aa327) and partly of intron 10 and exon 11. In contrast, the amplimer of around 400 bp from oligo-dT-generated cDNA consisted of exons 9 and 10.

### 2.6. Case Presentation of Patients Initiating Metreleptin Treatment

#### 2.6.1. Patient 027

This is a 61-year-old woman who was misdiagnosed as having type 1 diabetes. She was referred to our center due to uncontrolled diabetes despite high doses of insulin and the coexistence of severe insulin resistance and decreased body mass index (BMI: 19.4 kg/m^2^). Her age of presentation was 29 years, and for the previous 30 years, she had been treated with multiple daily insulin injections with high insulin requirements (5 IU/kg/day) together with 2 g/day of metformin. However, she constantly displayed poor glycemic control. She also presented dyslipidemia from the age of 35 years with raised cholesterol levels and severe hypertriglyceridemia despite intense lipid-lowering therapy together with restricted fat intake and hypertension from the age of 5 years. She presented peripheral diabetic neuropathy, nephropathy, peripheral artery disease (intermittent claudication with a right carotid endarterectomy at the age of 52 years old) and hypertrophic cardiomyopathy. She also reported polycystic ovarian syndrome and had never conceived. The examination revealed lipoatrophy of her upper and lower limbs and gluteal area, facial fat and abdominal prominence. She also had phlebectasia, hirsutism and cervical acanthosis nigricans. She was underweight with a BMI of 19.40 kg/m^2^ (weight: 46 kg, height: 154 cm). At the time of the first evaluation, her biochemical measurements were as follows: 10% glycated hemoglobin (HbA1C), 132 mg/dL total cholesterol (of which high-density lipoprotein (HDL-C) was 25 mg/dL) and severe hypertriglyceridemia at 2.919 mg/dL. Her plasma leptin concentration was close to zero (0.43 ng/mL). An abdominal ultrasound confirmed hepatic steatosis, and transient elastography (a fibroscan) detected liver stiffness of 18 Kpa. The genetic analysis revealed a novel heterozygous mutation in the PPARG gene (c470A > G, p. Glu157Gly, exon3), which is associated with a 78.6% probability of causing FPLD3. Metreleptin was then initiated at 5 mg once daily on top of the current lipid and diabetes management. Glycemic control and hypertriglyceridemia improved within three months of treatment evidenced by a decrease in HbA1C from 10 to 8.7% and a reduction in TG from a baseline value of 2.919 to 242 mg/dL. This improvement was sustained one year after treatment with metreleptin at the same dose (HbA1C: 8%, TG: 185 mg/dL). The insulin doses were reduced from more than 5 to 2.22 IU/kg/day. A reduction in appetite and weight loss following metreleptin treatment were reported. This case report has been described in detail previously in the literature [11].

#### 2.6.2. Patient 034

This is a 35-year-old woman who was referred to our center due to uncontrolled diabetes from the age of 22 years despite intensive antidiabetic medication (inj. liraglutide 1.8 mg, metformin 2 mg, pioglitazone 15 mg) with high doses of insulin (insulin degludec 70 IU). She had presented hypertension from the age of 16 years, hypertriglyceridemia, polycystic ovarian syndrome and hypertrophic cardiomyopathy. Her examination revealed she had a BMI of 30.50 kg/m^2^ (weight: 90 kg, height: 172 cm); lipoatrophy of the upper and lower limbs and the gluteal area; trunk, neck and facial fat; and abdominal prominence. She also had hirsutism and cervical acanthosis nigricans. At the time of the first evaluation, her biochemical measurements revealed glycated hemoglobin (HbA1C) level of 8.9%, a total cholesterol level of 279 mg/dL (of which low-density lipoprotein (LDL-C) was 145 mg/dL), hypertriglyceridemia at 431 mg/dL and albuminuria (ACR: 80.13 mg/gr). Her plasma leptinconcentration was 10.7 ng/mL. An abdominal ultrasound confirmed hepatic steatosis, and abdominal magnetic resonance imaging (MRI) revealed a liver percentage fat fraction of 18.06%. The genetic analysis did not reveal mutations in the PPARG and LMNA genes. Initially, her antidiabetic treatment was modified with the discontinuation of pioglitazone due to the increase in her weight and the initiation of 25 mg empagliflozin daily. Metreleptin was then initiated at 5 mg once daily on top of the current lipid and diabetes management. Her glycemic control and hypertriglyceridemia improved within three months of treatment evidenced by a decrease in HbA1C from 8.9 to 7.8% and a reduction in TG from a baseline value of 431 to 182 mg/dL. This improvement was sustained one year after treatment with metreleptin in the same dose (HbA1C: 8.2%, TG: 249 mg/dL). After one year of metreleptin treatment, a reduced liver fat content was observed (her MRI-estimated liver percentage fat fraction was reduced from 18.9 to 16.8%). Her insulin doses were reduced from 0.7 IU/kg/day to 0.4 IU/kg/day.

#### 2.6.3. Patient 036

This is a 19-year-old male who was diagnosed as having type 1 diabetes. He was referred to our center due to uncontrolled diabetes despite high doses of insulin and the coexistence of severe insulin resistance and a decreased body mass index (BMI: 15.54 kg/m^2^). The age of diabetes presentation was 15 years. The patient was treated with multiple daily insulin injections with high insulin requirements (1 IU/Kg/day). A clinical examination revealed clinical signs of lipoatrophy of the upper and lower limbs with the coexistence of muscular atrophy and weakness. He also presented clinical characteristics of progeroid syndrome: a beaked nose, thin lips and mandibular hypoplasia. At the time of the first evaluation, his biochemical measurements revealed a glycosylated hemoglobin (HbA1C) level of 8.7% and plasma leptin concentration of 4 ng/mL. We performed a glucagon stimulation test which revealed residual insulin secretion (fasting C-peptide: 5.09 ng/mL, 6 min after 1 mg of glucagon infusion C-peptide: 8.17 ng/mL). An abdominal ultrasound did not reveal hepatic steatosis. The genetic analysis did not reveal mutations in the AGPAT2, BSCL2, CAV1, CAVIN1, PLIN1, LIPE, AKT2, LMNA or PPRAG genes. Metreleptin was initiated at 5 mg once daily on top of the patient’s current diabetes management. His glycemic control improved within three months of treatment evidenced by a decrease in HbA1C from 8.7% to 6.6%. The insulin treatment was discontinued. This improvement was sustained one year after treatment with metreleptin at the same dose (HbA1C: 6.7%). A reduction in appetite and weight loss following the metreleptin treatment were reported, and the metreleptin treatment was discontinued.

#### 2.6.4. Patient 037

This is a 42-year-old woman who has had severe hypertriglyceridemia from the age of 22 years old despite intense lipid-lowering therapy with 145 mg fenofibrate and omega-3 fatty acids as well as restricted fat intake. She was hospitalized at our center due to acute pancreatitis. She had also presented diabetes from the age of 28 years with poor glycemic control despite intensive antidiabetic medication with multiple daily insulin injections in high doses (1.5 IU/kg/day) together with 2 g metformin/day. She has presented hypertension since adolescence, diabetic neuropathy, high cholesterol levels and hypothyroidism. She also reported infertility issues with eight miscarriages. An examination revealed she had a BMI of 30.16 kg/m^2^ (weight: 89 kg, height: 172 cm), lipoatrophy of the upper and lower limbs and the gluteal area, facial fat and abdominal prominence. She also had cervical acanthosis nigricans. At the time of the first evaluation, her biochemical measurements revealed a glycated hemoglobin (HbA1C) level of 10.1% and hypertriglyceridemia at 3390 mg/dL. Her plasma leptin concentration was 5.8 ng/mL. An abdominal ultrasound confirmed hepatic steatosis, transient elastography (a fibroscan) detected liver stiffness of 6.9 Kpa and abdominal magnetic resonance imaging (MRI) revealed a liver percentage fat fraction of 16.09%. The genetic analysis did not reveal mutations in the AGPAT2, BSCL2, CAV1, CAVIN1, PLIN1, LIPE, AKT2, PPARG or LMNA genes. After noting her poor glycemic control, her antidiabetic treatment was intensified with 25 mg empagliflozin daily. Metreleptin was then initiated at 5 mg once daily on top of the patient’s current lipid and diabetes management. Her glycemic control and hypertriglyceridemia improved within three months of treatment evidenced by a decrease in HbA1C from 10.9 to 7.8% and a reduction in TG from a baseline value of 3390 to 267 mg/dL. Her insulin doses were reduced from 1.5 IU/day to 0.5 IU/day.

#### 2.6.5. Patient 038

This is a 52-year-old woman who was referred to our center due to uncontrolled diabetes from the age of 28 years despite intensive antidiabetic medication (inj. semaglutide 1 mg, metformin 2 mg, dapagliflozin 10 mg) with high doses of insulin (1.6 IU/Kg/day). She also has presented hypertension from the age of 20 years and severe hypertriglyceridemia, despite intensive lipid-lowering therapy (fenofibrate 145 mg) together with restricted fat intake. Moreover, she reported polycystic ovarian syndrome and infertility issues. Her examination revealed she had a BMI of 22 kg/m^2^ (weight: 85 kg, height: 141 cm), lipoatrophy of the upper and lower limbs and the gluteal area, facial fat and abdominal prominence. At the time of the first evaluation, biochemical measurements revealed a glycated hemoglobin (HbA1C) level of 8.9% and hypertriglyceridemia at 589 mg/dL. Her plasma leptin concentration was 15 ng/mL. An abdominal ultrasound confirmed hepatic steatosis, transient elastography (a fibroscan) detected liver stiffness of 6.8 Kpa and abdominal magnetic resonance imaging (MRI) revealed a liver percentage fat fraction of 18.79%. The genetic analysis did not reveal mutations in the AGPAT2, BSCL2, CAV1, CAVIN1, PLIN1, LIPE, AKT2, PPARG or LMNA genes. Metreleptin was then initiated at 5 mg once daily on top of the patient’s current lipid and diabetes management. Her glycemic control and hypertriglyceridemia improved within three months of treatment evidenced by a decrease in HbA1C from 8.9 to 7.9% and a reduction in TG from a baseline value of 589 to 129 mg/dL. Her insulin doses were reduced from 1.6 IU/kg/day to 0.3 IU/day.

#### 2.6.6. Patient 039

This is a 44-year-old woman who was diagnosed with type 1 diabetes with negative autoimmune-related type 1 diabetes antibodies (tyrosine phosphatase antibodies and glutamic acid decarboxylase antibodies). She was referred to our center due to uncontrolled diabetes, which she had presented since she was 25 years old. She was being treated with multiple daily insulin injections (insulin glargine: 26 IU and insulin aspart: SF:1/15, CR:1/30), but constantly displayed poor glycemic control due to high glucose variability with the presence of retinopathy as a microvascular complication of diabetes. She had presented NAFLD and dyslipidemia from a young age, and she was under treatment with 20 mg rosuvastatin. The examination revealed she had a BMI of 26 kg/m^2^ (weight: 64 kg, height: 157 cm), lipoatrophy of the lower limbs and gluteal area, and abdominal prominence. Regarding her family history, her mother had presented a lipodystrophy phenotype and diabetes at a young age. She had had a fatal myocardial infraction at the age of 55 years old. Her sister had also displayed a lipodystrophy phenotype and diabetes from a young age as well as premature menopause. At the time of the first evaluation, the biochemical measurements revealed a glycated hemoglobin (HbA1C) level of 7.7%. The plasma leptin concentration was 3.5 ng/mL. Transient elastography (fibroscan) detected liver stiffness of 3.8 Kpa. Two-dimensional echocardiography revealed left ventricular hypertrophy and an ejection fraction of 60%. The genetic analysis did not reveal mutations in the AGPAT2, BSCL2, CAV1, CAVIN1, PLIN1, LIPE, AKT2, PPARG or LMNA genes. Metreleptin was initiated at 5 mg once daily on top of the patient’s current lipid and diabetes management. Glycemic control improved within three months of treatment evidenced by the improvement in glucose variability, the substantial reduction in hypoglycemic episodes and the decrease in HbA1C from 7.7 to 6.1% with the discontinuation of insulin aspart and the decrease in the dose of insulin glargine from 26 IU to 10 IU. The metabolic and anthropometric parameters of the patients who received metreleptin treatment are summarized in Table 4.

## 3. Discussion

In this study, genetic testing for mutations in the LMNA gene in a large cohort of patients with FPLD did not result in any of the already known pathogenic mutations, but it revealed three likely pathogenic mutations, along with various changes in other exons and especially introns, whose pathogenicity and subsequent role in the patients’ phenotype remain unclear. In addition, it was shown that treatment with metreleptin in specific FPLD patients led to substantial improvements in terms of glycemic and lipidemic control, an effect which was sustained at the 12-month follow-up.

Taking into account the fact that the known pathogenic variants in LMNA and PPARG account for only 50% of the FPLD cases worldwide, it becomes evident that not only other genes per se, but also other still unknown mutations in these two genes are involved in the pathogenesis of the disease [5]. The great majority of the variants in the LMNA gene correspond to missense changes in various exons, with the main ones affecting Arginine 482; however, nonsense changes, deletions and duplications have also been reported in rare cases. We have sequenced the exons and the flanking intronic areas, but we did not screen for mutations in regulatory regions and all introns, so we cannot disregard the fact that important mutations may be present in these areas. Therefore, it is important that, in our study, a new possibly pathogenic variant was discovered, causing a substitution of glutamic acid and aspartic acid, which could account for the patients’ phenotype. Despite the fact that both Glu and Asp are negatively charged amino acids, even a small change in the side chain could result in a protein with altered functionality. The main finding of the genetic results in our study, however, is the fact that in most of the patients, no significant changes were detected at the exon level, and any mutations that led to changes at the protein level were not associated with a lipodystrophic phenotype. On the contrary, various changes were detected at the intron level, especially in introns 7 and 10, whose clinical significance is considered unknown. Whether these changes could be responsible for the overt lipodystrophic phenotype of the patients was seriously doubted until recently, considering the fact that introns are non-coding areas of DNA; however, a rising number of reports over the last years have suggested a role for introns in the pathophysiology of the disease. One of the first reports was published by Al-Shali et al. [12], in which no coding sequence mutations in either LMNA or PPARG were found in a female FPLD patient; instead, a novel A > G mutation at position −14 of intron B upstream of PPARG exon 1 within the promoter of the PPARγ4 isoform was discovered. Whether this mutation alone could have caused overt lipodystrophy in this patient could not be established, despite the clear association between PPARG and fat metabolism, and it was suggested that a second genetic defect could be required to express the lipodystrophic phenotype. The important role of introns in disease pathogenesis was also pointed out by Morel et al. [13], who carried out a mutation analysis of LMNA in two sisters with a severe form of FPLD2 (Dunnigan type). The sisters were heterozygous for a novel G > C mutation at the intron 8 consensus splice donor site, which resulted in a truncated, dysfunctional lamin A isoform. A splicing mutation was also revealed by Horn et al. [14], who identified a novel heterozygous de novo splice site mutation c.8226þ1G > T, affecting the last intron of the fibrillin 1 (FBN1) gene in a patient with progeroid and lipodystrophic features, and similar results have been shown in reports concerning cases of generalized lipodystrophy [15,16]. Alterations in splicing sites are well established as significant changes that could result in non-functional proteins. The plethora of data regarding intronic polymorphisms and lipodystrophic phenotypes could lead to the hypothesis that alterations in intronic regions could play a role, though yet to be clarified. This “multiple hit” theory was proposed again by Paolacci et al. [17], who discovered multiple POLR3A variants in deep intronic regions in patients with progeria.

Apart from the aforementioned data, which imply that the intronic changes detected in our cohort may play an active role in the phenotypic expression of the patients, the exonic mutations with yet unknown clinical significance could also account for the defective phenotype. In fact, lipodystrophies are syndromes which are characterized by a profound genetic, allelic and phenotypic heterogeneity. The expression and activity of the LMNA gene could be affected by single-nucleotide polymorphisms (SNPs) both inside (coding) and outside (non-coding) the gene locus, such as SNPs affecting the LMNA binding or the binding of transcription factors, as in the case of the PPARG gene [6]. In addition, the phenotypic expression of a specific mutation is subject both to epigenetic factors, such as DNA methylation, and environmental factors, such as age, gender and diet. As a result, the same mutation can lead to various phenotypes in different individuals, even within the same family. Therefore, the polymorphisms detected in our cohort could be of clinical significance, a hypothesis which, however, would require a wider genetic panel to be analyzed according to the “multiple hit” theory before any solid conclusions could be reached.

Regarding the effects of metreleptin treatment in the five eligible FPLD patients, a significant reduction in HbA1c and triglycerides was shown within the first 3 months of treatment, and, even more importantly, these favorable effects were sustained after 1 year. Τhese results are generally consistent with those of previous reports on patients with FPLD, although some discrepancies exist. In a study exclusively on patients with FPLD, metreleptin treatment led to sustainable decreases in HbA1c (−0.6%) and TGs (−20.8%) after 12 months [10]. The results were even more impressive in a subgroup of patients with more serious metabolic derangement (HbA1c ≥ 6.5% and TGs ≥ 500 mg/dL), in which the mean reductions in HbA1c and TGs were −0.9% and −37.4%, respectively, even after the multivariate analysis included concomitant medications, implying that these improvements could be directly due to metreleptin. In another study by Vatier et al. [18], HbA1c and TGs were significantly decreased by 1% and 27%, respectively, in nine patients with FPLD after 1 year of metreleptin treatment; their results were similar to those of a cohort of lipodystrophic patients with severe baseline metabolic abnormalities, in which mean reductions of −0.88% and −119.8 mg/dL were noted in HbA1c and TGs, respectively, at the 1-year follow-up [19]. In a study by Adamski et al. [20], the effects of metreleptin in FPLD patients were again favorable at 12 months, with an HbA1c reduction of −0.61% and a TG reduction of −28.7%, respectively; however, it was noted that these results were seriously more modest compared to the effects of the treatment in GL patients. This difference has also been observed in other cohorts. In a cohort of 29 lipodystrophic individuals, statistically significant reductions in HbA1c were shown both in patients with LMNA and with PPARG variants (−0.5% and −1.5%, respectively), but a substantial reduction in TGs was only noted in the LMNA group; however, it was pointed out that both groups were more likely to experience clinically relevant triglyceride (≥30%) or HbA1c (≥1%) reductions if they had a worse baseline metabolic profile (triglycerides ≥ 500 mg/dL or HbA1c ≥ 8%) [9]. Even more impressively, the metabolic benefit of metreleptin on glycemic control has not always been consistent among patients with FPLD. In a study by Mosbah et al. [21], despite a significant reduction in TGs by approximately 25% after 12 months, no significant difference was observed in HbA1clevelsin FPLD patients, again in contrast to the results in the GL group. In a study by Simha et al. [22], who compared the response to metreleptin between Dunnigan-type patients with severe vs moderate hypoleptinemia, serum triglycerides were reduced to a similar extent in both groups, but without a corresponding improvement in the HbA1c values. In a similar cohort of Dunnigan-type patients, the initial decrease of −65% in the TG values after 4 months of treatment continued to a statistically significant decrease of −43% after 12 months; however, HbA1c was only mildly, and not significantly, reduced by −0.4% [23].

The reasons behind these discrepancies have not yet been fully elucidated. The small number of patients and the heterogeneous causes of FPLD could account for methodological differences which could obscure the treatment effects on metabolic endpoints and their interpretation. In our study, the response to metreleptin was homogeneous among the patients regardless of their previous treatment regimen; however, in some reports, it has been shown that insulin therapy at baseline is associated with poorer improvements in HbA1c, implying perhaps that the hypoglycemic action of metreleptin is dependent on preserved beta cell function [20]. The metabolic improvements indicated in our cohort were also independent of any demographic, anthropometric or laboratory parameters, including baseline serum leptin levels. This was the case in a study by Simha et al. [22], in which the effects on TGs and HbA1c were similar between the two groups with either severe or moderate hypoleptinemia. On the other hand, in a prospective, open-label study in which 31 FPLD patients were enrolled, metreleptin improved HbA1c and triglycerides in all FPLD subgroups except baseline triglycerides that were less than 500 mg/dL, HbA1c less than 8% or endogenous leptin greater than 4 ng/mL [24]. Similarly, endogenous leptin levels failed to predict metreleptin responders in a study by Sekizkardes et al. [9], but as mentioned above, a less favorable metabolic profile was associated with higher improvements in terms of TGs and HbA1c. In a study by Mosbah et al. [21], the most beneficial effects of metreleptin on glucose homeostasis were shown in the patients with the lowest leptin levels. In general, the endogenous leptin cut-off point that can predict response to metreleptin treatment is still a matter of debate, considering the questionable sensitivity and, therefore, comparability of the commercially available leptin assays. The same applies to baseline metabolic parameters that can function as response predictors, although it seems that a more adverse profile has an inverse association with the magnitude of metabolic improvement. It seems that the early diagnosis of lipodystrophy and the prompt initiation of targeted treatment could delay the progress of the disease. A genetic analysis could be conducted at any age; however, according to the international guidelines, the diagnosis of lipodystrophy is based on the clinical phenotype and the presence of family history, and treatment should not be delayed due to a possible negative genetic result.

## 4. Materials and Methods

### 4.1. Study Population

This is a prospective observational study which included patients who were admitted to the Lipodystrophy Outpatient Clinic of Attikon University Hospital, Chaidari, Greece, between 1 January 2014 and 31 December 2021. In total, 39 patients were identified to fulfil the clinical criteria of FPLD, defined by the American Association of Clinical Endocrinologists (AACE) as follows [25]: a gradual loss of subcutaneous adipose tissue in the extremities and/or gluteal regions with fat accumulation in intra-abdominal areas or in the face and neck, occurring around or shortly after puberty; the presence of acanthosis nigricans, polycystic ovaries syndrome (PCOS), severe hypertriglyceridemia or a history of pancreatitis due to hypertriglyceridemia, diabetes with severe insulin resistance (requirement for high doses of insulin, i.e ≥200 U/day or ≥2 U/kg/day), non-alcoholic fatty liver disease (NAFLD), prominent muscularity and phlebomegaly in the extremities, hyperphagia, secondary hypogonadism in males or primary/secondary amenorrhea in females, and/or a family history of a similar phenotype of lipodystrophy. The exclusion criteria included the presence of autoimmune disease, current antiretroviral therapy, presence of panniculitis, a history of allogenic hematopoietic stem cell transplantation during childhood, alcohol abuse, Cushing syndrome and a history of C3 glomerulopathy.

Anthropometric measurements including weight, height, waist circumference and body mass index (BMI) were recorded in all patients at baseline. A complete medical history was obtained, and all concomitant medications were recorded. The study protocol was approved by the ethics committee of the hospital, and a written informed consent form was obtained by all the patients enrolled.

### 4.2. Biochemical Measurements

Metabolic parameters were obtained at baseline for all patients and at 3 and 12 months after initiation of treatment for patients receiving metreleptin. Serum leptin was measured by RIA using a commercial kit before initiation of metreleptin. The biochemical parameters that were evaluated included glycosylated hemoglobin (HbA1c), triglycerides (TGs), total cholesterol (TC), high-density lipoprotein cholesterol (HDL-C), low-density lipoprotein cholesterol (LDL-C), aminotransferases, urea and creatinine.

### 4.3. Genetic Studies

Venous blood samples (in EDTA collection tubes) were collected from all patients. In all patients, the genetic analysis included a sequence and deletion/duplication analysis of LMNA and PPRARG genes. In four patients (P36–P39), the genetic analysis included the following genes: AGPAT2, BSCL2, CAV1, CAVIN1, PLIN1, PPARG, LIPE, LMNA, AKT2, exon and exon–intron junctions. The LMNA exons, including the splice site regions, were amplified in 11 segments (10) and PPARG exons in seven segments (11) from 50 ng of genomic DNA using the PCR and exon-specific primers pairs (available on request). The resulting PCR products were analyzed in an agarose gel and purified using a PCR purification kit (Qiagen, Hilden, Germany). The purified PCR products were sequenced using dye-terminator chemistry and an ABI 3730xl DNA analyzer. Sequence variants were verified by manually inspecting the chromatograms of both the wild-type and mutated products. For the investigation of alternative splicing, total RNA was isolated from whole blood (Macherey-Nagel) and was reverse-transcribed using random hexamers and oligo dT primers in separate reactions (using Invitrogen, Waltham, MA, USA, First strand cDNA synthesis kit). cDNA was subjected to 35 cycles of PCR using 4 different pairs of primers (7F-8R, 6F-9R, 5F-10R and 5F-11R, with numbers corresponding to exons). A total of 5 μL was electrophoresed on 1.8% agarose gel, and PCR products were sequenced directly (Applied Biosystems, Waltham, MA, USA, BigDye terminator v3.1). Total RNA from a donor without the mutation was used as control.

### 4.4. Imaging Studies

Hepatic steatosis was evaluated via upper-abdominal ultrasonography and magnetic-resonance-imaging-derived proton density fat fraction (MRI-PDFF). Cardiac performance was evaluated via two-dimensional echocardiography.

### 4.5. Metreleptin Treatment

Patients eligible to receive metreleptin (Myalepta^®^, AmrytPharmaceuticals, Dublin, Ireland) were those who fulfilled the therapeutic indications as these are described in the drug SmPC [7].

## 5. Conclusions

In this cohort of FPLD patients from a large referral center in Greece, we have shown the presence of mutations both in exons, which are different from the ones with an already established association with the disease, and in introns, which, based on recent literature, might also contribute to the final amino acid products and the phenotype of the patient. This is the main novelty of our study, as the role of introns in the clinical manifestations of various diseases has not been elucidated and has been an object of research in recent years. In addition, we have confirmed the well-known favorable results of metreleptin treatment in FPLD patients, which are independent from any baseline parameters and were sustained at the one-year follow-up. More large-scale studies are necessary to shed light on the genetic and allelic heterogeneity of the disease, along with the possible demographic, body and laboratory parameters which could predict treatment response and, therefore, assist in selecting the most suitable candidates for treatment.

## Figures and Tables

**Table 1:** The clinical characteristics of the patients in the study.

Patient Number	Age	Sex	Reason for Medical Consultation	Acanthosis Nigricans	Clinical Lipoatrophy	Fat Deposition	Family History	Comorbidities
01	35	F	NASH	+	Lower limbs Gluteal area	Abdomen Face Neck Trunk	Mother: T2DM, Lipodystrophy phenotype, coronary artery disease Son: Lipodystrophy phenotype	Prediabetes PCOS Dyslipidemia Hypertension
02	63	F	T2DM	+	Lower limbs Gluteal area	Abdomen Face Neck Trunk	Daughter:NASH, Lipodystrophy phenotype, dyslipidemia, Hypertension Father: T2DM, Mother: Τ2DM	Coronary artery disease Hypertension Dyslipidemia NASH
03	55	F	NASH	+	Upper and Lower limbs Gluteal area	Abdomen Face Neck Trunk	Mother: Τ2DM	Neurotonic dystrophy Prediabetes Hypertriglyceridemia
04	48	F	NASH	-	Upper and Lower limbs	Abdomen	Mother: T2DM	Prediabetes Hypertriglyceridemia
05	45	M	NASH	-	Upper and lower limbs	Abdomen	Mother: Lipodystrophy phenotype, NASH Father: T2DM	Hypertension
06	49	F	T2DM	+	Lower Limbs	Abdomen Face Trunk	Mother: T2DM, Dyslipidemia	Dyslipidemia
07	45	M	Hypetriglyceridia	-	Upper and Lower limbs	Abdomen Face	Father: T2DM, Dyslipidemia	Hypertension NAFLD
08	52	M	NASH	+	Lower limbs Gluteal area	Abdomen Face	Mother: Lipodystrophy phenotype, NASH	Hypertension Prediabetes
09	40	F	T2DM	+	Lower Limbs Gluteal area	Abdomen Trunk Face Neck	Four siblings: T2DM	Dyslipidemia Hypothyroidism Depression
10	42	F	T2DM	+	Lower Limbs Gluteal area	Abdomen Trunk Face Neck	Brother:coronary artery disease	Hypertension Hypertriglycetidemia NAFLD Premature menopause
11	55	F	T2DM	+	Upper and Lower Limbs Gluteal Area	Abdomen	Sister: T2DM, Lipodystrophy phenotype	T2DM Dyslipidemia
12	35	F	Gestational Diabetes Mellitus	+	Upper and Lower Limbs	Abdomen Trunk	Mother: T2DM Father: T2DM	Hypertension NASH
13	43	F	NASH	-	Lower Limbs Gluteal Area	Abdomen	Mother: T2DM Brother: Τ2DM NASH	Prediabetes
14	50	M	Hypertriglyceridemia	+	Upper and Lower Limbs Gluteal Area	Abdomen	Father: T2DM	Hypertension NASH
15	55	F	T2DM	+	Upper and Lower Limbs Gluteal Area	Abdomen	Father: T2DM Paternal Aunt: Τ2DM	NAFLD Depression Hypertriglyceridemia
16	39	F	T1DM	+	Upper and Lower Limbs Gluteal area	Abdomen Trunk Face Neck	Sister: Τ2DM, NASH	PCOS Nephropathy
17	58	F	T2DM	+	Lower Limbs Gluteal area	Abdomen Trunk Face Neck	Father: Τ2DM Five Siblings: T2DM Mother:T2DM	Myocardial Infraction Hashimoto Heart Failure Dyslipidemia Hypertension
18	45	F	T2DM	+	Lower Limbs Gluteal area	Abdomen	Father: T2DM, Hypertension	Hypertension Hypertriglyceridemia
19	48	F	NASH	-	Lower Limbs	Abdomen	Mother: Dyslipidemia	Hypertriglyceridemia
20	53	F	NASH	+	Lower Limbs Gluteal area	Abdomen Trunk Face	Father: Coronary Artery Disease Paternal	Prediabetes Hypertension Dyslipidemia
21	55	M	NASH T2DM	+	Upper and Lower Limbs Gluteal area	Abdomen Trunk	Father: T2DM, Hypertension	Hypertension
22	46	F	T2DM	+	Upper and Lower Limbs Gluteal area	Abdomen Trunk Face Neck	Father: Coronary Artery Disease Paternal Aunt: T2DM Paternal Grandmother: T2DM	Myocardial Infraction ,PCI Dyslipidemia
23	44	F	Hypertrigyceridemia	+	Upper and Lower Limbs Gluteal area	Abdomen	Mother: Dyslipidemia Coronary artery disease	PCOS
24	43	F	Hypertriglyridemia	+	Upper and Lower Limbs Gluteal area	Abdomen Trunk Face Neck	Daughter: Lipodystrophy phenotype Mother: Dyslipidemia Coronary artery disease PCOS	PCOS Multiple pancreatitis Hypertension
25	58	M	T2DM	+	Upper and Lower Limbs Gluteal area	Abdomen	Father: T2DM Paternal aunt: T2DM	Hypertension Dyslipidemia
26	66	F	T2DM	+	Upper and Lower Limbs gluteal area	Abdomen Face Neck	Mother: T2DM Father: T2DM Paternal aunt: T2DM	Hypertension Dyslipidemia Peripheral Neuropathy
27	61	F	T1DM	*+*	Upper and Lower Limbs Gluteal area	Abdomen Face		Dyslipidemia Nephropathy Peripheral diabetic neuropathy Peripheral artery disease Hypertrophic cardiomyopathy PCOS Infertility
28	61	M	T2DM	*-*	Upper and Lower Limbs	Abdomen	Son: Hypertension, T2DM, Dyslipidemia	Dilated Cardiomyopathy Dyslipidemia Hypertension Kidney Failure
29	49	F	T2DM	+	Upper and Lower Limbs	Abdomen	Mother:Hypertension Two Siblings: Hypertension Paternal aunt: NASH	Hypertension Hypertriglyceridemia NASH
30	42	F	T2DM	+	Upper and Lower Limbs	Abdomen Face	Mother: Dyslipidemia Coronary artery disease	Hypertriglyceridemia PCOS Hypertension NASH
31	45	F	T2DM	+	Upper and Lower Limbs	Trunk Abdomen Face Neck	Father: Τ2DM	Hypertension NASH Hypertriglyceridemia
32	28	F	T1DM	*+*	Lower Limbs	Abdomen Face Neck Trunk	Father: T2DM	PCOS Psychotic Disorder Hypertension Amenorrhea NAFLD
33	55	M	T2DM	*+*	Lower Limbs	Abdomen	Father: T2DM, Dyslipidemia	Hypertension
34	35	F	T2DM	*+*	Lower Limbs Gluteal area	Trunk Abdomen Face Neck	Father: Τ2DM	Hypertension Hypertensive cardiomyopathy Infertility Hypothyroidism Dyslipidemia NASH PCOS
35	60	F	T2DM	*+*	Lower Limbs, Gluteal area	Trunk Abdomen Face	Mother:T2DM, Dyslipidemia, Coronary artery Disease, Lipodystrophy phenotype Maternal Aunt: T2DM, Dyslipidemia Lipodystrophy phenotype	Dyslipidemia(severe hypertriglyceridemia) NAFLD Peripheral Artery Disease
36	19	M	T1DM	*-*	Upper and Lower limbs Face	-	-	
37	42	F	Severe Hypertriglyceridemia	*+*	Upper and Lower Limbs Gluteal area	Trunk Abdomen Face Neck	Father:T2DM Mother:T2DM, Hypertension, Dyslipidemia Sister:Lipodystrophy phenotype	T2DM Diabetic Neuropathy Hypertension Hospitalization due to acute pancreatitis Hypothyroidism
38	52	F	T2DM	*-*	Upper and Lower Limbs Gluteal area	Abdomen Face	Mother:T2DM Father:T2DM	Hypertension Hypertriglyceridemia PCOS Infertility
39	44	F	T1DM	-	Lower limbs Gluteal area	Abdomen	Mother: Lipodystrophy phenotype, T2DM, Myocardial Infraction Sister: Lipodystrophy phenotype, DM, premature menopause	NAFLD Dyslipidemia Retinopathy

NASH: non-alcoholic steatohepatitis; T2DM: type 2 diabetes mellitus; PCOS: polycystic ovarian syndrome; NAFLD: non-alcoholic fatty liver disease; T1DM: type 1 diabetes mellitus.

**Table 2:** The anthropometric and biochemical characteristics of the patients in the study.

Anthropometric and Metabolic Parameters	Mean	±SD
BMI (Kg/m^2^)	29.10	4.71
WC (cm)	105.66	14.96
HbA1c (%)	7.23	1.41
ALT (U/L)	33.71	26.52
AST (U/L)	31.51	18.52
Chol (mg/dL)	200.25	61.50
HDL (mg/dL)	35.55	8.72
LDL (mg/dL)	112.68	33.15
TGs (mg/dL)	403.33	670.38
Bun (mg/dL)	29.56	12.19
Cr (mg/dL)	0.81	0.25

BMI: body mass index; WC: waist circumference; HbA1c: glycosylated hemoglobin; ALT: alanine aminotransferase; AST: aspartate aminotransferase; Chol: cholesterol; HDL: high-density cholesterol; LDL: low-density cholesterol; TGs: triglycerides; BUN: blood urea nitrogen; Cr: creatinine.

**Table 3:** Results of the genetic testing of lipodystrophic patients. VUS: variant of unknown significance.

	Gene	Exon/ Intron	Nucleotide Change	Zygosity	Amino Acid Change	Significance of Genetic Variance
01	LMNA	Intron 7	c.1381-127_1381-126delAG	Heterozygous	-	VUS
02	LMNA	Intron 7	c.1381-127_1381-126delAG	Heterozygous	-	VUS
03	LMNA	Intron 9	c.1608+143A>G	Heterozygous	-	VUS
04	LMNA	-	-	-	-	-
05	LMNA	Exon 10	c.1698C>T	Homozygous	pHis566His	Polymorphism
06	LMNA	-	-	-	-	-
07	LMNA	Intron 7	c.1381-127_1381-126delAG	Heterozygous	-	VUS
08	LMNA	Intron 3 Intron3	IVS3 +123G>T IVS3-154G>T	Heterozygous Heterozygous	- -	Polymorphism Polymorphism
09	LMNA	-	-	-	-	-
10	LMNA	-	-	-	-	-
11	LMNA	-	-	-	-	-
12		Intron 2	c.513+175T>C	Heterozygous		VUS
		Exon 5	c.861T>C	Heterozygous	p.Ala287Ala	Polymorphism
	LMNA	Intron 5	c.937-83G>T	Heterozygous		VUS
		Intron 6	c.1157+16G>A	Heterozygous		VUS
		Exon 7	c.1338T>C	Heterozygous		Polymorphism
		Intron 7	c.1380+142G>A	Heterozygous		VUS
		Intron 8	c.1489-41C>T	Heterozygous		VUS
13	LMNA	Exon 10	c.1698 C>T		pHis566His	Polymorphism
14	LMNA	Exon 10	c.1698 C>T		pHis566His	Polymorphism
15		Intron 2	c.513+175T>C	Heterozygous	-	VUS
	LMNA	Exon5	c.861T>C	Heterozygous	p.Ala287Ala	Polymorphism
		Intron 5	c.937-83G>T	Heterozygous	-	VUS
		Intron 6	c.1157+16G>A	Heterozygous	-	Polymorphism
		Exon 7	c.1338T>C	Heterozygous	p.Asp466Asp	VUS
		Intron 7	c.1489-41C>T	Heterozygous	-	VUS
16	LMNA	Exon 10	c.1698C>T	Heterozygous	pHis566His	Polymorphism
17	LMNA	-	-	-	-	-
18	LMNA	Exon 10	c.1698C>T	Heterozygous	pHis566His	Polymorphism
19	LMNA	-	-	-	-	-
20	LMNA	Exon 10	c.1698C>T	Heterozygous	pHis566His	Polymorphism
21	LMNA	-	-	-	-	-
22		Intron 2	c.513+175T>C	Heterozygous	*-*	
	LMNA	Exon 5	c.861T>C	Heterozygous	p.Ala287Ala	
		Intron 5	c.937-83G>T	Heterozygous		
		Exon 7	c.1338T>C	Heterozygous	p.Asp446Asp	
		Intron 7	c.1380+142G>A	Heterozygous		
		Intron 8	c.1489-41C>T	Heterozygous		
		Exon 10	c.1698C>T	Heterozygous	p.His566His	Polymorphism
23	LMNA	Exon 10	c.1698C>T	Heterozygous	pHis566His	Polymorphism
24		Intron 2	c.513+175C>T	Homozygous	-	VUS
		Intron 3	c.639+56G>T	Heterozygous	-	VUS
	LMNA	Exon 5	c.861T>C	Omozygous	p.Ala287Ala	Polymorphism
		Intron 5	c.937-83G>T	Omozygous	-	VUS
		Intron 6	c.1157+16G>A	Omozygous	-	VUS
		Exon 7	c.1338T>C	Omozygous	pAsp446Asp	Polymorphism
		Intron 7	c.1489-41	Omozygous	-	VUS
		Intron 11	c.1969-1G>A		-	Mutation???
25	LMNA	Exon 10	c.1698C>T	Heterozygous	pHis566His	Polymorphism
26	LMNA	Exon 10	c.1698C>T	Heterozygous	*pHis566His*	Polymorphism
27	LMNA	Exon 10	c.1698C>T	Heterozygous	p.His566His	Polymorphism
	PPARG	Exon 10	c. 470 A>G	Heterozygous	PGlu157Gly	Likely pathogenic variable
28		Intron 1	c.329+71C>G	Heterozygous	*-*	
		Exon 5	c.861T>C	Heterozygous	*p.Ala287Ala*	
	LMNA	Intron 6	c.1157+16G>A	Heterozygous	*-*	
		Exon 7	c.1338T>C	Heterozygous	*p.Asp446Asp*	
		Intron 8	c.1489-41C>T	Heterozygous		
29	LMNA	Exon 10	c.1698C>T	Heterozygous	pHis566His	Polymorphism
30	LMNA	Exon 4	c.786G>T	Heterozygous	pGlu262Asp	Possible pathogenic mutation
		Exon 10	c.1698C>T	Heterozygous	-	Polymorphism
31	LMNA	Exon 10	c.1698C>T	Heterozygous	pHis566His	Polymorphism
32	LMNA	-	-	-	*-*	-
33	LMNA	-	-	-	*-*	-
34	LMNA	-	-	-	*-*	-
	PPARG	-	-	-	*-*	-
35	LMNA	-	-	-	*-*	-
	PPARG	-	-			-
36	LMNA	-	-	-	*-*	-
	PPARG	-	-	-	*-*	-
37	LMNA	-	-	-	*-*	-
	PPARG	-	-	-	*-*	-
38	LMNA	-	-	-	*-*	-
	PPARG	-	-	-	*-*	-
39	LMNA	-	-	-	*-*	-
	PPARG	-	-	-	*-*	-

**Table 4:** Summary of the results over 12 months of metreleptin therapy.

	Patient 027			Patient 034			Patient 036			Patient 037			Patient 038			Patient 039		
	Bas	3 m	12 m	Bas	3 m	12 m	Bas	3 m	12 m	Bas	3 m	12 m	Bas	3 m1	12 m	Bas	3 m	12 m
HbA1C (%)	10	8.7	8	8.9	7.8	8.2	8.7	6.6	6.7	10.1	7.8	-	8.9	7.9	-	7.7	6.1	-
TG (mg/dL)	2919	242	185	431	182	249	157		91	3390	267	-	589	129	-	128	82	-
Total cholesterol (mg/dL)	132	137	106	279	211	165	143		154	442	157	-	271	108	-	108	155	-
HDL (mg/dL)	25	25	29	48	45	44	36		45	20	24	-	38	36	-	27	48	-
LDL (mg/dL)	-	64	46	145	130	72	76		91	-	80	-	-	46	-	55	91	-
ALT (mg/dL)	24	24	17	38	33	29	35		25	20	22	-	22	18.9	-	24	13	-
AST (mg/dL)	21	27	26	23	31	14	18		17	10	17	-	14	16.6	-	25	17	-
BUN (mg/dL)	49.6	26	39	27	26	28	39		136	14.8	17.3	-	28	21	-	27	28	-
Creatinine (mg/dL)	1.3	1.3	1.09	0.73	0.73	0.66	0.76		0.79	0.5	0.7	-	0.8	0.9	-	0.82	0.73	-
Liver fat fraction (%)	9.4	-	6.8	18.06	-	16.8	-	-	-	16.9		-	18.79	-	-	3.8	-	-
Liver Stiffness (Kpa)	18	-	18		-		-	-	-			-	6.8	-	-	3.8	-	-
BMI (Kg/m^2^)	19.40	-	18.98	30.50	28.81	27.11	15.54			30.16	29.1	-	22	20.7	-	26	17.47	-

## Data Availability

Study data are available upon reasonable request.
